# Randomized, placebo‐controlled, phase 3 study of perifosine combined with bortezomib and dexamethasone in patients with relapsed, refractory multiple myeloma previously treated with bortezomib

**DOI:** 10.1002/jha2.4

**Published:** 2020-04-06

**Authors:** Paul G. Richardson, Arnon Nagler, Dina Ben‐Yehuda, Ashraf Badros, Parameswaran N. Hari, Roman Hajek, Ivan Spicka, Hakan Kaya, Richard LeBlanc, Sung‐Soo Yoon, Kihyun Kim, Joaquin Martinez‐Lopez, Moshe Mittelman, Ofer Shpilberg, Paul Blake, Teru Hideshima, Kathleen Colson, Jacob P. Laubach, Irene M. Ghobrial, Merav Leiba, Moshe E. Gatt, Peter Sportelli, Michael Chen, Kenneth C. Anderson

**Affiliations:** ^1^ Jerome Lipper Center for Multiple Myeloma Research Dana‐Farber Cancer Institute Boston Massachusetts USA; ^2^ Chaim Sheba Medical Center Tel Hashomer Israel; ^3^ Hadassah University Hospital Jerusalem Israel; ^4^ Greenebaum Comprehensive Cancer Center University of Maryland Baltimore Maryland USA; ^5^ Department of Hematology/Oncology Medical College of Wisconsin Milwaukee Wisconsin USA; ^6^ Department of Hematooncology University Hospital, Ostrava, and Faculty of Medicine University of Ostrava Ostrava Czech Republic; ^7^ First Department of Medicine, Department of Hematology First Faculty of Medicine Charles University and General Hospital in Prague Prague Czech Republic; ^8^ Cancer Care Northwest Spokane Washington USA; ^9^ CIUSSS de l'est de l’île de Montréal University of Montreal Montreal Canada; ^10^ Department of Internal Medicine Seoul National University College of Medicine Seoul South Korea; ^11^ Sungkyunkwan University School of Medicine Samsung Medical Center Seoul South Korea; ^12^ Hospital Universitario 12 de Octubre CNIO Complutense University Madrid Spain; ^13^ Tel Aviv Sourasky Medical Center Tel Aviv Israel; ^14^ Institute of Hematology Assuta Medical Centers Tel Aviv and Ariel University Ariel Israel; ^15^ Aeterna Zentaris Frankfurt Germany; ^16^ Assuta Ashdod University Hospital Faculty of Health Sciences Ben‐Gurion University of the Negev Beer‐Sheba Israel; ^17^ Keryx Biopharmaceuticals Inc. New York New York USA; ^18^ TCM Groups Inc. Berkeley Heights New Jersey USA

**Keywords:** Akt inhibition, bortezomib, multiple myeloma, perifosine, proteasome inhibition

## Abstract

Perifosine, an investigational, oral, synthetic alkylphospholipid, inhibits signal transduction pathways of relevance in multiple myeloma (MM) including PI3K/Akt. Perifosine demonstrated anti‐MM activity in preclinical studies and encouraging early‐phase clinical activity in combination with bortezomib. A randomized, double‐blind, placebo‐controlled phase 3 study was conducted to evaluate addition of perifosine to bortezomib‐dexamethasone in MM patients with one to four prior therapies who had relapsed following previous bortezomib‐based therapy. The primary endpoint was progression‐free survival (PFS). The study was discontinued at planned interim analysis, with 135 patients enrolled. Median PFS was 22.7 weeks (95% confidence interval 16·0–45·4) in the perifosine arm and 39.0 weeks (18.3–50.1) in the placebo arm (hazard ratio 1.269 [0.817–1.969]; *P* = .287); overall response rates were 20% and 27%, respectively. Conversely, median overall survival (OS) was 141.9 weeks and 83.3 weeks (hazard ratio 0.734 [0.380–1.419]; *P* = .356). Overall, 61% and 55% of patients in the perifosine and placebo arms reported grade 3/4 adverse events, including thrombocytopenia (26% vs 14%), anemia (7% vs 8%), hyponatremia (6% vs 8%), and pneumonia (9% vs 3%). These findings demonstrate no PFS benefit from the addition of perifosine to bortezomib‐dexamethasone in this study of relapsed/refractory MM, but comparable safety and OS.

## 1 INTRODUCTION

The treatment of multiple myeloma (MM) has been transformed over the past two decades with the introduction of novel targeted agents, such as proteasome inhibitors, immunomodulatory drugs, and monoclonal antibodies, and as a result of our increasing understanding of the complex disease biology of MM and the signaling pathways of importance [1, 2, 3, 4, 5]. Proteasome inhibitors and immunomodulatory drugs have emerged as backbone therapeutics for MM treatment as a result of the substantial efficacy demonstrated alone and in combination in different disease settings [6]. This activity arises due to these mechanisms of action affecting multiple critical signaling pathways of importance [7, 8]. However, as MM progresses, patients can develop resistance to these and other commonly used agents. Therefore, an ongoing unmet need for patients with relapsed and/or refractory MM (RRMM) is for novel targeted agents that inhibit specific pathways of relevance and potentially synergize with or overcome resistance to the mechanisms of action of the proteasome inhibitors and the immunomodulatory drugs [9, 10, 11].

One of the pathways of interest for targeting in the treatment of MM has been the phosphoinositide‐3‐kinase (PI3K)/Akt signaling cascade, the activation of which is induced by interactions between MM cells and bone marrow stromal cells within the bone marrow microenvironment [12, 13]. The induction of this and other signaling pathways results in MM proliferation, survival, and drug resistance [13], making it a rational therapeutic target in MM. Perifosine is an investigational, oral, synthetic alkylphospholipid that inhibits or modifies signal transduction pathways of relevance in MM including PI3K/Akt, nuclear factor‐κB, and c‐Jun N‐terminal kinase (JNK) cascades [13]. Perifosine demonstrated potent anti‐MM activity in preclinical studies [14, 15, 16, 17, 18], including enhanced cytotoxicity in combination with bortezomib based on synergism between mechanisms of action [19].

Consequently, perifosine was investigated clinically in MM and demonstrated encouraging activity in patients with MM when combined with bortezomib and lenalidomide [20, 21]. In a phase 1/2 study in 73 patients with RRMM, perifosine in combination with bortezomib, with or without added dexamethasone, resulted in a rate of minimal response or better of 41%, including rates of 65% in patients who had relapsed following prior bortezomib treatment and 32% in bortezomib‐refractory patients [21]. Based on these promising early‐phase study results, we conducted a randomized, double‐blind, placebo‐controlled phase 3 study to evaluate the benefit of adding perifosine to bortezomib‐dexamethasone in MM patients who had previously relapsed after a bortezomib‐based regimen.

## 2 METHODS

### 2.1 Patients

Between March 2010 and March 2013, relapsed and/or refractory patients aged ≥18 years with a confirmed diagnosis of MM, who had measurable disease (serum M‐protein > 0.5 g/dL and/or > 200 mg/24‐h urinary M‐protein excretion) and an Eastern Cooperative Oncology Group performance status of 0–2, were enrolled at 48 study sites in the United States, Israel, Spain, South Korea, Canada, Russia, the Czech Republic, France, Ireland, and Slovakia. Patients were required to have received 1–4 prior anti‐myeloma therapies, including at least two 21‐day cycles of either single‐agent bortezomib or bortezomib in combination with other agents, and patients had to have relapsed following their prior bortezomib‐based therapy, with progression occurring >60 days after last dose (ie, patients could not be refractory to prior bortezomib‐containing regimens). Patients could have relapsed following or have been refractory to other non‐bortezomib‐based therapies. Patients also required adequate hematological (platelets ≥75 × 10^9^/L, absolute neutrophil count ≥0.5 × 10^9^/L, hemoglobin ≥8.0 g/dL), renal (creatinine ≤3.0 mg/dL), and hepatic (total bilirubin ≤1.5 × upper limit of normal) function. Patients were excluded if they had previously received treatment with perifosine or an experimental proteasome inhibitor.

### 2.2 Study design

This was a double‐blind, placebo‐controlled, randomized phase 3 study (NCT01002248). Patients were randomized in a 1:1 ratio to receive perifosine 50 mg orally, once‐daily, or matching placebo, plus intravenous or subcutaneous bortezomib 1.3 mg/m^2^ on days 1, 4, 8, and 11 and oral dexamethasone 20 mg on days 1, 2, 4, 5, 8, 9, 11, and 12, in 21‐day treatment cycles until disease progression. Randomization was stratified according to number of prior lines of therapy (1 vs > 1) and disease status after last prior therapy (refractory or relapsed with a treatment‐free interval [TFI] of <6 vs ≥6 months). Dose reductions and delays were permitted for the management of toxicity.

The primary endpoint was progression‐free survival (PFS). Secondary endpoints included overall response rate, overall survival (OS), and safety. Tertiary endpoints included perifosine population pharmacokinetics and evaluation of treatment effects on electrocardiogram (ECG) parameters. Serum and urine protein electrophoresis were performed by a central laboratory at the start of each 21‐day treatment cycle to assess disease status until confirmed disease progression. Response or progression determined according to parameters other than serum and urine M‐protein or determined by local laboratory readings were adjudicated by an independent reviewer blinded to treatment arms. All responses were assessed using modified European Group for Blood and Marrow Transplantation criteria [22] and International Myeloma Working Group Uniform Criteria [23]. Survival status was assessed every 3 months. Toxicity was recorded throughout the study across both arms and through 30 days following the last dose of treatment; severity of adverse events (AEs) was assessed using the National Cancer Institute's Common Terminology Criteria for AEs version 3.0, with attribution assessed locally and centrally as part of standard monitoring practice for safety.

### 2.3 Statistical analyses

Enrolment of 450 patients was planned in order to provide 265 events of disease progression or death for the final statistical analysis of the primary endpoint, PFS. Two formal nonbinding interim analyses were planned by the independent Data and Safety Monitoring Board (DSMB) to assess efficacy at the time of approximately 30% and 60% of the total PFS events having occurred, respectively, that is when approximately 80 and 160 PFS events had been observed. The results of the first planned interim analysis are reported herein, which was performed when 80 PFS events had been observed across the two arms. Following the recommendation of the DSMB, the study was discontinued at this interim analysis (March 12, 2013) due to absence of benefit in the primary endpoint, limited study logistics, and enrolment challenges, which had resulted in very slow patient accrual and thereby limited the sample size.

The primary analysis was performed using the intent‐to‐treat population and included only those progression events defined by a computer algorithm and, when required, an independent reviewer. Progression determined by the investigator alone was not considered a progression event. Hypothesis testing between the two treatment arms was performed using a log‐rank test with an overall two‐sided .05 level of significance via a model including treatment effects. For each treatment arm, the median duration of PFS and the proportion of patients alive and progression‐free at 6 and 12 months were estimated using the Kaplan–Meier method. For each estimate, a 95% confidence interval (CI) was reported. The hazard ratio (HR) and its 95% CI were also reported. The secondary endpoint of OS was analyzed similarly to PFS. Other data were summarized using descriptive statistics.

## 3 RESULTS

### 3.1 Patients

**FIGURE 1:**
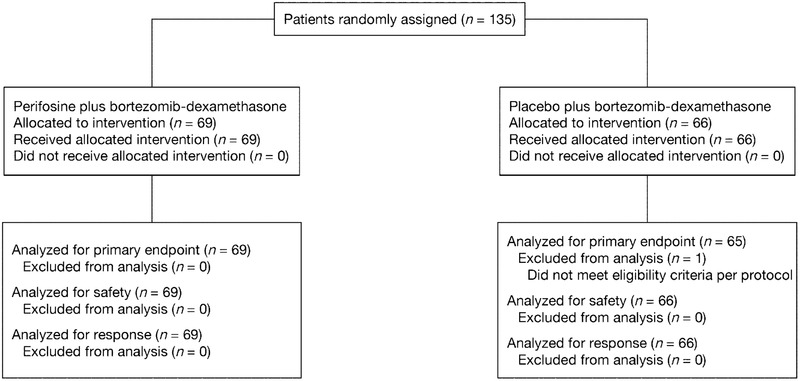
CONSORT diagram of patient disposition through the study

At the data cut‐off for the first interim analysis (March 12, 2013), a total of 135 patients had been enrolled and randomized – 69 to the perifosine arm and 66 to the placebo arm (Figure 1). Patient demographics and disease characteristics at randomization were similar between treatment arms (Table 1), except for a numerically higher proportion of patients aged <65 years in the perifosine arm versus the placebo arm (61% vs 42%). Prior treatment exposure and relapsed/refractory status were balanced between arms.

**TABLE 1:** Patient demographics and disease characteristics at randomization

	Perifosine + Vd, n = 69	Placebo + Vd, n = 66
Age <65/≥65 years, n (%)	41 (61)/26 (39)^*^	28 (42)/38 (58)
Male/female, n (%)	41 (60)/27 (40)^†^	37 (56)/29 (44)
Race, n (%)	n = 68^†^	n = 66
White	58 (85)	54 (82)
Asian	6 (9)	7 (11)
Black or African American	4 (6)	5 (8)
ECOG PS, n (%)^‡^	n = 56	n = 57
0	32 (57)	31 (54)
1	17 (30)	24 (42)
2	7 (13)	2 (4)
Myeloma subtype, n (%)	n = 65^§^	n = 66
IgG	43 (66)	45 (68)
IgA	10 (15)	14 (21)
IgM	0	2 (3)
IgD	1 (2)	0
Other	11 (17)	5 (8)
Prior therapy and disease status (stratification), n (%)	n = 69	n = 66
1 line, refractory^#^	2 (3)	2 (3)
>1 line, refractory	15 (22)	12 (18)
1 line, relapse, TFI < 6 months	3 (4)	4 (6)
>1 line, relapse, TFI < 6 months	16 (23)	16 (24)
1 line, relapse, TFI ≥ 6 months	6 (9)	7 (11)
>1 line, relapse, TFI ≥ 6 months	27 (39)	25 (38)
Disease status at randomization, n (%)	n = 67*	n = 66
Relapsed	55 (82)	54 (82)
Refractory	12 (18)	12 (18)

* Data missing for two patients.

† Data missing for one patient.

‡ Data missing for 13 and 9 patients in the perifosine and placebo arms, respectively.

§ Data missing for four patients.

# Patients not refractory to bortezomib‐based component of prior line of therapy.

Abbreviations: ECOG PS, Eastern Cooperative Oncology performance status; Ig, immunoglobulin; TFI, treatment‐free interval; Vd, bortezomib‐dexamethasone.

### 3.2 Efficacy

**FIGURE 2:**
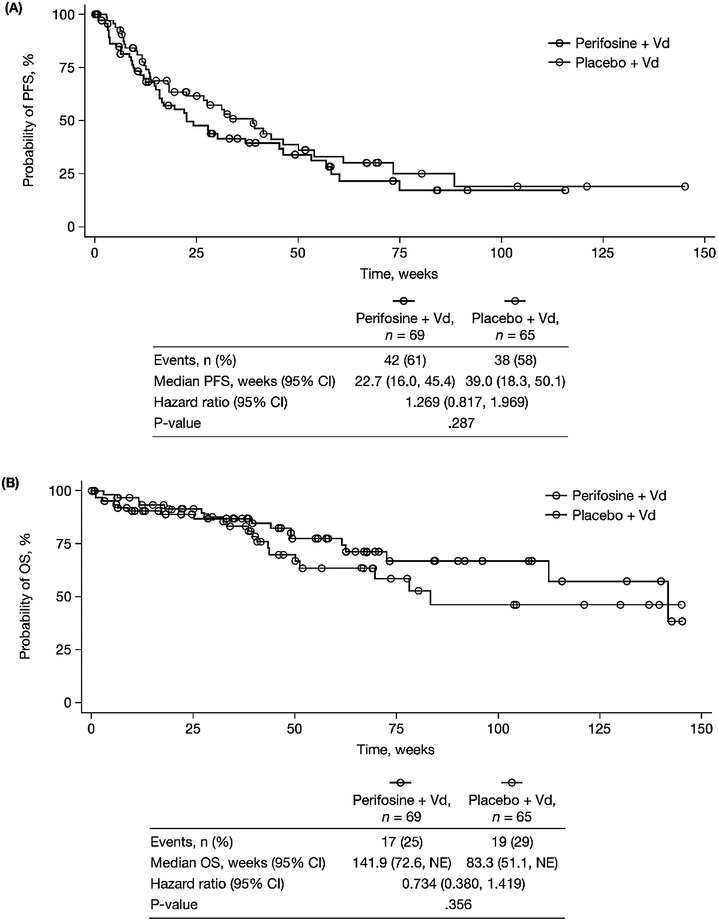
Kaplan‐Meier plots of (A) progression‐free survival and (B) overall survival with perifosine or placebo plus bortezomib‐dexamethasone in patients with relapsed, refractory MM. [CI, confidence interval; NE, not estimable; OS, overall survival; PFS, progression‐free survival; Vd, bortezomib‐dexamethasone.]

The primary endpoint of PFS was assessed in the intent‐to‐treat population, with the exception of one patient in the placebo arm. This patient was not evaluable for time‐to‐event outcomes; subsequent to randomization, the patient was found not to meet eligibility criteria based on M‐protein levels required by the protocol, and so they were excluded from the analyses of PFS and OS. At the time of data cutoff for this first planned interim analysis, a total of 42 patients in the perifosine arm and 38 patients in the placebo arm had progressed or died, with 27 patients in each arm remaining alive and progression‐free after a median follow‐up of approximately 28 and 31 weeks, respectively. The median PFS was 22.7 weeks (95% CI 16.0–45.4) in the perifosine arm and 39.0 weeks (18.3–50.1) in the placebo arm; the HR for PFS in the perifosine arm relative to the placebo arm was 1.269 (0.817–1.969; *P* = .287). The Kaplan–Meier distributions for PFS are shown in Figure 2A.

The overall response rates were 20% (14 patients) and 27% (18 patients) in the perifosine and placebo arms, respectively (Table 2). An additional 18 and 11 patients, respectively, achieved a minimal response or stable disease, giving clinical benefit rates (stable disease or better) of 46% and 44%.

**TABLE 2:** Best response to treatment

Best response, n (%)	Perifosine + Vd, n = 69	Placebo + Vd, n = 66
Overall response rate	14 (20.3)	18 (27.3)
Complete response	2 (2.9)	0
Very good partial response	4 (5.8)	5 (7.6)
Partial response	8 (11.6)	13 (19.7)
Clinical benefit rate	32 (46.4)	29 (43.9)
Minimal response	5 (7.2)	6 (9.1)
Stable disease	13 (18.8)	5 (7.6)
Progressive disease	9 (13.0)	7 (10.6)
Unevaluable	5 (7.2)	4 (6.1)
Missing	23 (33.3)	26 (39.4)

At the time of data cut‐off, a total of 17 patients in the perifosine arm and 19 patients in the placebo arm had died, with 52 and 46 patients in the respective arms remaining alive after a median follow‐up of approximately 48 and 40 weeks, respectively. The median OS was 141.9 weeks (95% CI 72.6–not estimable) in the perifosine arm and 83.3 weeks (51.1–not estimable) in the placebo arm; the HR for OS in the perifosine arm relative to the placebo arm was 0.734 (0.380–1.419; *P* = ·356). The Kaplan–Meier distributions for OS are shown in Figure 2B.

### 3.3 Safety

All 135 patients were evaluable for safety. In total, 60 (87%) of patients in the perifosine arm and 50 (75.8%) of patients in the placebo arm reported at least one AE; the most common events are summarized in Table 3. Among these patients, 43 (62%) and 38 (58%) reported at least one grade ≥3 AE; the most common individual grade 3/4 AE events in the perifosine versus placebo arms included thrombocytopenia (26% vs 14%), anemia (7% vs 8%), hyponatremia (6% vs 8%), and pneumonia (9% vs 3%; Table 3). Rates of grade 3 neuropathy AEs were limited, including two patients (3%) on each arm with peripheral neuropathy and only one patient (2%) on the placebo arm having a grade 3 AE of peripheral sensory neuropathy; no grade 4 neuropathy AEs were reported. However, four patients (6%) on the perifosine arm and one patient (2%) on the placebo arm discontinued treatment due to an AE of neuropathy or neuralgia. Overall, 13 (19%) and 9 (14%) patients on the perifosine and placebo arms, respectively, discontinued treatment due to an AE. Other AEs resulting in treatment discontinuation on the perifosine arm were pneumonia (n = 2), and thrombocytopenia, oral pain/toothache, meningioma, bronchitis, asthenia, diarrhea, and ocular hyperemia (each n = 1), and on the placebo arm were respiratory failure/sepsis, nausea, congestive heart failure, fatigue, diarrhea/asthenia/thrombocytopenia, back pain, abdominal pain, and muscular weakness/pain in extremity (each n = 1).

**TABLE 3:** Summary of most frequently reported all‐grade (reported in ≥10% of patients in either arm) and grade 3/4 adverse events (reported in ≥5% of patients in either arm)

	Perifosine + Vd, n = 69		Placebo + Vd, n = 66	
	All	Grade 3/4	All	Grade 3/4
Any AE	60 (87)	42 (61)^*^	50 (76)	36 (55)^*^
Thrombocytopenia	24 (35)	18 (26)	15 (23)	9 (14)
Fatigue	19 (28)	4 (6)	20 (30)	2 (3)
Diarrhea	18 (26)	2 (3)	19 (29)	2 (3)
Anemia	15 (22)	5 (7)	12 (18)	5 (8)
Neuropathy peripheral	14 (20)	2 (3)	12 (18)	2 (3)
Nausea	14 (20)	1 (1)	8 (12)	2 (3)
Insomnia	13 (19)	2 (3)	8 (12)	1 (2)
Asthenia	10 (14)	1 (1)	11 (17)	3 (5)
Hyperglycemia	9 (13)	3 (4)	7 (11)	3 (5)
Constipation	9 (13)	0	18 (27)	0
Upper respiratory tract infection	9 (13)	0	10 (15)	0
Pneumonia	8 (12)	6 (9)	4 (6)	2 (3)
Hyponatremia	7 (10)	4 (6)	7 (11)	5 (8)
Hypophosphatemia	6 (9)	2 (3)	8 (12)	3 (5)
Peripheral edema	6 (9)	0	12 (18)	1 (2)
Neutropenia	4 (6)	1 (1)	7 (11)	5 (8)

Abbreviations: AE, adverse event; SOC, system organ class; Vd, bortezomib‐dexamethasone.

* In addition, one patient on the perifosine arm and two patients on the placebo arm had grade 5 AEs, as described in the text.

A total of four patients (6%) on the perifosine arm and three patients (5%) on the placebo arm died during the study reporting period due to reasons other than disease progression, including respiratory infection, suicide, sudden death (treatment‐emergent grade 5 AE), and myocardial infarction (each n = 1) on the perifosine arm, and respiratory failure/sepsis (treatment‐emergent grade 5 AE), congestive heart failure, and pneumococcal sepsis (treatment‐emergent grade 5 AE) (each n = 1) on the placebo arm.

## 4 DISCUSSION

The findings from the first planned interim analysis of this randomized, double‐blind, placebo‐controlled, phase 3 study demonstrated no benefit in terms of the primary endpoint, PFS, with the addition of perifosine to bortezomib‐dexamethasone in patients with RRMM who had previously relapsed following bortezomib‐based treatment. Based on these data and the recommendation of the DSMB, and associated with concerns regarding slow accrual resulting in a limited sample size and regarding resource constraints and study logistics, the study was discontinued following this first planned interim analysis. Thus, with limited follow‐up, further interpretation of these findings is restricted. In particular, OS data were immature at the time of this analysis, with only 25% and 29% of patients in the perifosine and placebo arms, respectively, having died. Median OS was numerically longer in the perifosine arm, but these data were based on the tail‐ends of the Kaplan–Meier distribution curves and must therefore be interpreted with caution.

The response rates reported in the present study appeared low compared to data from the previous phase 1/2 study of perifosine plus bortezomib‐dexamethasone in RRMM [21]; in the previous study, an overall response rate of 45% was reported, compared to 20% in the present study, and all patients achieved stable disease or better, compared to 46%. However, these previous data were based on a cohort of only 20 bortezomib‐relapsed patients who had received a median of 4 prior therapies, suggesting ongoing sensitivity to drug therapy in these patients. By contrast, the data from the present study suggest that the enrolled patients had relatively resistant disease, with almost a quarter stratified as refractory to other, non‐bortezomib‐based prior therapies. Additionally, the discrepancy between the phase 2 and phase 3 experiences may have been due to the small number of patients in the previous open‐label phase 2 study; this may not have been optimal for subsequent phase 3 exploration – a larger, randomized phase 2 study or an adaptive study design may have been preferable and may potentially have provided response data more similar to those reported from the present study. It is also notable that the response rate in the placebo arm (27%) appeared lower compared to data from a phase 2 study of bortezomib ± dexamethasone retreatment in patients with relapsed MM (40%) [24].

It is of interest to assess the overall response and clinical benefit rates in both arms in the context of the high proportion of unevaluable patients and patients missing response data (40.5% and 45.5% in the perifosine and placebo arms, respectively); if these patients were to be omitted from the calculations of overall response and clinical benefit rates, the rates would be similar to those previously reported for perifosine plus bortezomib‐dexamethasone in bortezomib‐relapsed RRMM patients and bortezomib ± dexamethasone retreatment in patients with relapsed MM (overall response rate of 34.1% and 50.0%, and clinical benefit rate of 78.0% and 80.6%, in the perifosine and placebo arms, respectively). Nevertheless, these findings suggest the acute antitumor activity of the triplet combination in bortezomib‐relapsed patients was primarily driven by bortezomib‐dexamethasone, a hypothesis supported by data from a previous multicenter phase 2 study of perifosine ± dexamethasone in RRMM patients after a median of 4 prior lines of therapy [25], in which single‐agent perifosine had modest activity and perifosine + dexamethasone resulted in an overall response rate of 12.9% and a clinical benefit rate of 38.7%. While the prior phase 1/2 study of perifosine plus bortezomib‐dexamethasone in RRMM suggested some antitumor activity or resensitizing effect of perifosine in bortezomib‐refractory patients (with an overall response rate of 13.2% and a clinical benefit rate of 32.1%) [21], such patients were not included in the present study.

A limitation of the present study is that, due to the dates between which it was conducted, a number of now‐commonly collected prognostic parameters were not recorded, including cytogenetics; thus, it is not possible to determine whether the patients in the study presented with an elevated rate of poor prognostic features, such as high‐risk cytogenetic abnormalities, which could potentially explain the poor response rates and outcomes seen in both arms. Further, no correlative studies related to the PI3K/Akt signaling cascade were prospectively planned; such analyses may have helped identify a patient population who did benefit from the combination [26].

Reflecting the previous phase 1/2 study of perifosine plus bortezomib‐dexamethasone [21] the triplet regimen appeared tolerable at the selected dose of perifosine; indeed, it might be queried, given the limited activity seen with the triplet regimen, whether the perifosine dose was optimal in this study, given that no dose‐limiting toxicities were reported using a 100 mg dose of perifosine in the previous phase 1/2 study [21]. However, as noted in that report, toxicity was generally greater in this higher‐dose cohort, and this impacted the duration of treatment; thus, the 50 mg dose was selected for the phase 2 component of the previous study and for the present study [21]. In the present study, no specific safety concerns were observed in the perifosine arm compared with the placebo arm. Common grade 3/4 AEs included thrombocytopenia, pneumonia, and anemia, which were also among the most common grade 3/4 AEs in the previous study [21]. Overall, the low rates of treatment discontinuation and the convenience of the oral approach of the triplet supported the real‐world strategy underlying the combination's development [27], as well as the equipoise in the design, given the favorable performance of the control group and the OS data in the experimental arm subsequent to interim analysis.

Subsequent to the discontinuation of this study, perifosine is no longer being investigated as a potential novel targeted agent for patients with RRMM. Reflecting these findings, a similar lack of OS benefit was seen with the addition of perifosine to capecitabine in patients with metastatic colorectal cancer in the phase 3 X‐PECT study [28]. Nevertheless, the PI3K/Akt signaling cascade remains a rational target of interest in MM [29, 30, 31] and multiple other cancers [32, 33, 34, 35], with several Akt inhibitors in ongoing clinical development either alone or in combination regimens [32, 36, 37, 38, 39], and preclinical data demonstrating the validity of this mechanism of action with perifosine and other agents both in hematological malignancies and solid tumors [40, 41, 42, 43]. Meanwhile, additional therapeutic approaches targeted at other signaling cascades of known importance are being explored in RRMM [9, 44], warranting further evaluation.

In conclusion, perifosine at the dose tested did not improve response rates or outcomes in combination with bortezomib‐dexamethasone in patients with RRMM who have relapsed following previous bortezomib‐based treatment. Although the rational combination of Akt pathway inhibition with perifosine and proteasome inhibition with bortezomib demonstrated synergistic anti‐MM activity in preclinical investigation [19], this was not reflected in the phase 3 clinical setting, despite earlier‐phase clinical studies showing promise.

## AUTHOR CONTRIBUTIONS

P.G.R. was the principal investigator and takes primary responsibility for the paper; P.G.R., A.N., D.B.‐Y., A.B., P.N.H., R.H., I.S., H.K., R.L.B., S.‐S.Y., K.K., J.M.‐L., M.M., O.S., K.C., J.P.L., I.M.G., M.L., M.E.G., and K.C.A. collected data and/or recruited patients; P.G.R., A.N., P.B., T.H., P.S., and K.C.A. designed the study. P.S. and M.C. participated in the statistical analysis; P.G.R., P.S., and M.C. co‐ordinated preparation and analysis of the dataset; P.G.R. and P.S. interpreted the data and prepared the manuscript. All authors reviewed and approved the manuscript.

## CONFLICT OF INTEREST

P.G.R. received research support from Oncopeptides, Celgene, Takeda, and Bristol‐Myers Squibb and served as advisory committee member for Karyopharm, Oncopeptides, Celgene, Takeda, Amgen, Janssen, and Sanofi. P.N.H. received research funding from Takeda and Celgene, and served as a consultant for Takeda, Celgene, Bristol‐Myers Squibb, Janssen, Spectrum, Pharmacyclics, and Kite. R.H. received consulting fees, honoraria, and research funding from Amgen, Takeda, Celgene, Janssen, Abbvie, Novartis, PharmaMar, and BMS. H.K. served as a consultant and also served on a Speakers Bureau for Takeda. J.M.‐L. received research funding from BMS, Janssen, Novartis, and Celgene, and served as a consultant for Janssen, Novartis, Celgene, and Roche. P.B. was an employee of Aeterna Zentaris. K.C. served as a consultant for Celgene. I.M.G. served as a consultant for GSK, Sanofi, Janssen, Takeda, Celgene, Karyopharm, AbbVie, GNS, Cellectar, Medscape, Genentech, Adaptive, BMS, Aptitude, Curio Science, and Oncopeptides, and served as an advisor for GSK, AbbVie, and BMS. P.S. was an employee and had equity interest in Keryx Biopharmaceuticals. M.C. served as a consultant for Keryx Biopharmaceuticals. K.C.A. served as a consultant for Celgene, Millennium Pharmaceuticals, Bristol Myers Squibb, Janssen, Gilead, and Sanofi, and was a scientific founder of Oncopep and C4 Therapeutics.

## References

[1] Bianchi G , Anderson KC . Understanding biology to tackle the disease: multiple myeloma from bench to bedside, and back. CA Cancer J Clin. 2014;64:422–44. 25266555 10.3322/caac.21252

[2] Goldschmidt H , Ashcroft J , Szabo Z , Garderet L . Navigating the treatment landscape in multiple myeloma: which combinations to use and when? Ann Hematol. 2019;98:1–18. 10.1007/s00277-018-3546-8 PMC6334731 30470875

[3] Hari P . Recent advances in understanding multiple myeloma. Hematol Oncol Stem Cell Ther. 2017;10:267–71. 28633036 10.1016/j.hemonc.2017.05.005

[4] Kumar SK , Rajkumar V , Kyle RA , van Duin M , Mateos MV , Gay F , et al. Multiple myeloma. Nat Rev Dis Primers. 2017;3:17046. 28726797 10.1038/nrdp.2017.46

[5] Richter J , Jagannath S . Society of hematologic oncology state of the art update and next questions: multiple myeloma. Clin Lymphoma Myeloma Leuk. 2018;18:693–702. 30287199 10.1016/j.clml.2018.09.003

[6] Kumar SK , Callander NS , Alsina M , Atanackovic D , Biermann JS , Castillo J , et al. NCCN Guidelines Insights: Multiple Myeloma, Version 3. 2018. J Natl Compr Canc Netw. 2018;16:11–20. 29295877 10.6004/jnccn.2018.0002

[7] Gandolfi S , Laubach JP , Hideshima T , Chauhan D , Anderson KC , Richardson PG . The proteasome and proteasome inhibitors in multiple myeloma. Cancer Metastasis Rev. 2017;36:561–84. 29196868 10.1007/s10555-017-9707-8

[8] Holstein SA , McCarthy PL . Immunomodulatory drugs in multiple myeloma: Mechanisms of action and clinical experience. Drugs. 2017;77:505–20. 28205024 10.1007/s40265-017-0689-1 PMC5705939

[9] Ocio EM , Richardson PG , Rajkumar SV , Palumbo A , Mateos MV , Orlowski R , et al. New drugs and novel mechanisms of action in multiple myeloma in 2013: a report from the International Myeloma Working Group (IMWG). Leukemia. 2014;28:525–42. 24253022 10.1038/leu.2013.350 PMC4143389

[10] Szalat R , Munshi NC . Novel agents in multiple myeloma. Cancer J. 2019;25:45–53. 30694859 10.1097/PPO.0000000000000355 PMC6589825

[11] Varga C , Laubach J , Hideshima T , Chauhan D , Anderson KC , Richardson PG . Novel targeted agents in the treatment of multiple myeloma. Hematol Oncol Clin North Am. 2014;28:903–25. 25212889 10.1016/j.hoc.2014.07.001

[12] Harvey RD , Lonial S . PI3 kinase/AKT pathway as a therapeutic target in multiple myeloma. Future Oncol. 2007;3:639–47. 18041916 10.2217/14796694.3.6.639

[13] Richardson PG , Eng C , Kolesar J , Hideshima T , Anderson KC . Perifosine, an oral, anti‐cancer agent and inhibitor of the Akt pathway: mechanistic actions, pharmacodynamics, pharmacokinetics, and clinical activity. Expert Opin Drug Metab Toxicol. 2012;8:623–33. 22512706 10.1517/17425255.2012.681376 PMC4467022

[14] Cirstea D , Hideshima T , Rodig S , Santo L , Pozzi S , Vallet S , et al. Dual inhibition of akt/mammalian target of rapamycin pathway by nanoparticle albumin‐bound‐rapamycin and perifosine induces antitumor activity in multiple myeloma. Mol Cancer Ther. 2010;9:963–75. 20371718 10.1158/1535-7163.MCT-09-0763 PMC3096071

[15] David E , Sinha R , Chen J , Sun SY , Kaufman JL , Lonial S . Perifosine synergistically enhances TRAIL‐induced myeloma cell apoptosis via up‐regulation of death receptors. Clin Cancer Res.2008;14:5090–98. 18698026 10.1158/1078-0432.CCR-08-0016

[16] Gajate C , Mollinedo F . Edelfosine and perifosine induce selective apoptosis in multiple myeloma by recruitment of death receptors and downstream signaling molecules into lipid rafts. Blood. 2007;109:711–9. 17003375 10.1182/blood-2006-04-016824

[17] Hideshima T , Catley L , Yasui H , Ishitsuka K , Raje N , Mitsiades C , et al. Perifosine, an oral bioactive novel alkylphospholipid, inhibits Akt and induces in vitro and in vivo cytotoxicity in human multiple myeloma cells. Blood. 2006;107:4053–62. 16418332 10.1182/blood-2005-08-3434 PMC1895278

[18] Huston A , Leleu X , Jia X , Moreau AS , Ngo HT , Runnels J , et al. Targeting Akt and heat shock protein 90 produces synergistic multiple myeloma cell cytotoxicity in the bone marrow microenvironment. Clin Cancer Res. 2008;14:865–74. 18245550 10.1158/1078-0432.CCR-07-1299

[19] Hideshima T , Catley L , Raje N , Chauhan D , Podar K , Mitsiades C , et al. Inhibition of Akt induces significant downregulation of survivin and cytotoxicity in human multiple myeloma cells. Br J Haematol.2007;138:783–91. 17760810 10.1111/j.1365-2141.2007.06714.x

[20] Jakubowiak AJ , Richardson PG , Zimmerman T , Alsina M , Kaufman JL , Kandarpa M , et al. Perifosine plus lenalidomide and dexamethasone in relapsed and relapsed/refractory multiple myeloma: a phase i multiple myeloma research consortium study. Br J Haematol. 2012;158:472–80. 22640031 10.1111/j.1365-2141.2012.09173.x

[21] Richardson PG , Wolf J , Jakubowiak A , Zonder J , Lonial S , Irwin D , et al. Perifosine plus bortezomib and dexamethasone in patients with relapsed/refractory multiple myeloma previously treated with bortezomib: results of a multicenter phase I/II trial. J Clin Oncol. 2011;29:4243–49. 21990396 10.1200/JCO.2010.33.9788

[22] Blade J , Samson D , Reece D , Apperley J , Bjorkstrand B , Gahrton G , et al. Criteria for evaluating disease response and progression in patients with multiple myeloma treated by high‐dose therapy and haemopoietic stem cell transplantation. Myeloma Subcommittee of the EBMT. European Group for Blood and Marrow Transplant. Br J Haematol.1998;102:1115–23. 9753033 10.1046/j.1365-2141.1998.00930.x

[23] Durie BG , Harousseau JL , Miguel JS , Blade J , Barlogie B , Anderson K , et al. International uniform response criteria for multiple myeloma. Leukemia. 2006;20:1467–73. 16855634 10.1038/sj.leu.2404284

[24] Petrucci MT , Giraldo P , Corradini P , Teixeira A , Dimopoulos MA , Blau IW , et al. A prospective, international phase 2 study of bortezomib retreatment in patients with relapsed multiple myeloma. Br J Haematol. 2013;160:649–59. 23293914 10.1111/bjh.12198

[25] Richardson P , Lonial S , Jakubowiak A , Krishnan A , Wolf J , Densmore J , et al. Multi‐center phase II study of perifosine (KRX‐0401) alone and in combination with dexamethasone (dex) for patients with relapsed or relapsed/refractory multiple myeloma (MM): promising activity as combination therapy with manageable toxicity. Blood. 2007;110:1164.

[26] Gills JJ , Dennis PA . Perifosine: update on a novel Akt inhibitor. Curr Oncol Rep. 2009;11:102–10. 19216841 10.1007/s11912-009-0016-4 PMC6957247

[27] Richardson PG , San Miguel JF , Moreau P , Hajek R , Dimopoulos MA , Laubach JP , et al. Interpreting clinical trial data in multiple myeloma: translating findings to the real‐world setting. Blood Cancer J. 2018;8:109. 30413684 10.1038/s41408-018-0141-0 PMC6226527

[28] Bendell JC , Ervin TJ , Senzer NN , Richards DA , Firdaus I , Lockhart AC , et al. Results of the X‐PECT study: a phase III randomized double‐blind, placebo‐controlled study of perifosine plus capecitabine (P‐CAP) versus placebo plus capecitabine (CAP) in patients (pts) with refractory metastatic colorectal cancer (mCRC). J Clin Oncol. 2017;30:LBA3501.

[29] Keane NA , Glavey SV , Krawczyk J , O'Dwyer M . AKT as a therapeutic target in multiple myeloma. Expert Opin Ther Targets. 2014;18:897–915. 24905897 10.1517/14728222.2014.924507

[30] Ramakrishnan V , Kumar S . PI3K/AKT/mTOR pathway in multiple myeloma: from basic biology to clinical promise. Leuk Lymphoma. 2018;59:2524–34. 29322846 10.1080/10428194.2017.1421760

[31] Wang L , Lin N , Li Y . The PI3K/AKT signaling pathway regulates ABCG2 expression and confers resistance to chemotherapy in human multiple myeloma. Oncol Rep. 2019;41:1678–90. 30664164 10.3892/or.2019.6968 PMC6365707

[32] Brown JS , Banerji U . Maximising the potential of AKT inhibitors as anti‐cancer treatments. Pharmacol Ther. 2017;172:101–15. 27919797 10.1016/j.pharmthera.2016.12.001 PMC6143165

[33] Fensterle J , Aicher B , Seipelt I , Teifel M , Engel J . Current view on the mechanism of action of perifosine in cancer. Anticancer Agents Med Chem. 2014;14:629–35. 24628236 10.2174/1871520614666140309225912

[34] Guidetti A , Carlo‐Stella C , Locatelli SL , Malorni W , Mortarini R , Viviani S , et al. Phase II study of perifosine and sorafenib dual‐targeted therapy in patients with relapsed or refractory lymphoproliferative diseases. Clin Cancer Res. 2014;20:5641–51. 25239609 10.1158/1078-0432.CCR-14-0770

[35] O'Donnell JS , Massi D , Teng MWL , Mandala M . PI3K‐AKT‐mTOR inhibition in cancer immunotherapy, redux. Semin Cancer Biol. 2018;48:91–103. 28467889 10.1016/j.semcancer.2017.04.015

[36] Becher OJ , Gilheeney SW , Khakoo Y , Lyden DC , Haque S , De Braganca KC , et al. A phase I study of perifosine with temsirolimus for recurrent pediatric solid tumors. Pediatr Blood Cancer.2017;64. doi: 10.1002/pbc.26409. 28035748

[37] Becher OJ , Millard NE , Modak S , Kushner BH , Haque S , Spasojevic I , et al. A phase I study of single‐agent perifosine for recurrent or refractory pediatric CNS and solid tumors. PLoS One. 2017;12:e0178593. 28582410 10.1371/journal.pone.0178593 PMC5459446

[38] Kushner BH , Cheung NV , Modak S , Becher OJ , Basu EM , Roberts SS , et al. A phase I/Ib trial targeting the Pi3k/Akt pathway using perifosine: long‐term progression‐free survival of patients with resistant neuroblastoma. Int J Cancer. 2017;140:480–84. 27649927 10.1002/ijc.30440 PMC5118186

[39] Matsumoto K , Shichino H , Kawamoto H , Kosaka Y , Chin M , Kato K , et al. Phase I study of perifosine monotherapy in patients with recurrent or refractory neuroblastoma. Pediatr Blood Cancer. 2017;64. doi: 10.1002/pbc.26623. 28521076

[40] Kim MN , Ro SW , Kim DY , Kim da Y , Cho KJ , Park JH , et al. Efficacy of perifosine alone and in combination with sorafenib in an HrasG12V plus shp53 transgenic mouse model of hepatocellular carcinoma. Cancer Chemother Pharmacol. 2015;76:257–67. 26037205 10.1007/s00280-015-2787-7

[41] Le Grand M , Berges R , Pasquier E , Montero MP , Borge L , Carrier A , et al. Akt targeting as a strategy to boost chemotherapy efficacy in non‐small cell lung cancer through metabolism suppression. Sci Rep. 2017;7:45136. 28332584 10.1038/srep45136 PMC5362809

[42] Shen J , Xu L , Zhao Q . Perifosine and ABT‐737 synergistically inhibit lung cancer cells in vitro and in vivo. Biochem Biophys Res Commun, 2016;473:1170–76. 27073162 10.1016/j.bbrc.2016.04.035

[43] Zhang J , Hong Y , Shen J . Combination treatment with perifosine and MEK‐162 demonstrates synergism against lung cancer cells in vitro and in vivo. Tumour Biol. 2015;36:5699–706. 25697899 10.1007/s13277-015-3244-2

[44] Nijhof IS , van de Donk N , Zweegman S . Lokhorst HM . Current and new therapeutic strategies for relapsed and refractory multiple myeloma: an update. Drugs. 2018;78:19–37. 29188449 10.1007/s40265-017-0841-y PMC5756574

